# High-throughput avian molecular sexing by SYBR green-based real-time PCR combined with melting curve analysis

**DOI:** 10.1186/1472-6750-8-12

**Published:** 2008-02-12

**Authors:** Hsueh-Wei Chang, Chun-An Cheng, De-Leung Gu, Chia-Che Chang, San-Hua Su, Cheng-Hao Wen, Yii-Cheng Chou, Ta-Ching Chou, Cheng-Te Yao, Chi-Li Tsai, Chien-Chung Cheng

**Affiliations:** 1 Faculty of Biomedical Science and Environmental Biology, Kaohsiung Medical University, Kaohsiung, Taiwan; 2 Graduate Institute of Natural Products, College of Pharmacy, Kaohsiung Medical University, Kaohsiung, Taiwan; 3 College of Life Science, National Tsing Hua University, HsinChu, Taiwan; 4 Institute of Biomedical Sciences, National Chung Hsing University, Taichung, Taiwan; 5 Institute of Biotechnology, Chung Hwa College of Medical Technology, Tainan, Taiwan; 6 Institute of Ecology and Evolutionary Biology, National Taiwan University, Taipei, Taiwan; 7 Taiwan Endemic Species Research Institute, Nantou, Taiwan; 8 Institute of Biodiversity, National Cheng Kung University, Tainan, Taiwan; 9 Department of Life Sciences, National Chung Hsing University, Taichung, Taiwan

## Abstract

**Background:**

Combination of *CHD *(chromo-helicase-DNA binding protein)-specific polymerase chain reaction (PCR) with electrophoresis (PCR/electrophoresis) is the most common avian molecular sexing technique but it is lab-intensive and gel-required. Gender determination often fails when the difference in length between the PCR products of *CHD-Z *and *CHD-W *genes is too short to be resolved.

**Results:**

Here, we are the first to introduce a PCR-melting curve analysis (PCR/MCA) to identify the gender of birds by genomic DNA, which is gel-free, quick, and inexpensive. *Spilornis cheela hoya *(*S. c. hoya*) and *Pycnonotus sinensis *(*P. sinensis*) were used to illustrate this novel molecular sexing technique. The difference in the length of *CHD *genes in *S. c. hoya *and *P. sinensis *is 13-, and 52-bp, respectively. Using Griffiths' P2/P8 primers, molecular sexing failed both in PCR/electrophoresis of *S. c. hoya *and in PCR/MCA of *S. c. hoya *and *P. sinensis*. In contrast, we redesigned sex-specific primers to yield 185- and 112-bp PCR products for the *CHD-Z *and *CHD-W *genes of *S. c. hoya*, respectively, using PCR/MCA. Using this specific primer set, at least 13 samples of *S. c. hoya *were examined simultaneously and the Tm peaks of *CHD-Z *and *CHD-W *PCR products were distinguished.

**Conclusion:**

In this study, we introduced a high-throughput avian molecular sexing technique and successfully applied it to two species. This new method holds a great potential for use in high throughput sexing of other avian species, as well.

## Background

Real-time PCR (polymerase chain reaction) is a well-established method for RNA quantification [1,2] and genomic DNA analysis [3-6]. Several analytical methods have been employed to detect PCR products. The most popular, the TaqMan assay [7], is a hybridization-based method with high specificity, but it is relatively expensive. Another method is based on the double-stranded DNA binding ability [8,9] of fluorescent dye SYBR Green I. The amount of PCR products (amplicons) can be detected in real-time in both methods by measuring fluorescence levels from the cycle threshold (Ct). Both types of analyses are potentially fast and sensitive but the SYBR Green I dye is more cost-effective than the TaqMan probe.

Recently, SYBR green real-time quantitative PCR has been applied to investigate the mRNA expression in birds. For example, the dosage compensation of Z-linked gene expressions was reported [10,11]. However, to our knowledge, real-time PCR has not been applied in genomic DNA studies such as the molecular sexing of birds. In general, the molecular sexing of birds depends on the difference in the length of *CHD *genes when comparing a universal *CHD *primer pair P2/P8 [12]. Traditionally, a single *CHD-Z *band is found in males and two bands in females [12] after electrophoresis (female, ZW; male, ZZ) when analyzing *CHD-Z *and *CHD-W *genes. Since real-time PCR can monitor the PCR products quantitatively and robustly without the need for gel electrophoresis, the advantages of real-time PCR can be utilized to evaluate avian molecular sexing using the P2/P8 primers. However, SYBR green real-time PCR alone cannot distinguish the individual fluorescence values from different PCR products mixed in the same well. Therefore, this disadvantage of SYBR green real-time PCR alone hinders its utility in molecular sexing using *CHD*-based primers.

Fortunately, the fluorescence detection problem of SYBR green real-time PCR may be hurdled by subsequent coupling with SYBR green-based melting curve analysis (MCA). MCA was originally used to confirm PCR product identity and to differentiate between specific and non-specific PCR products [13]. Although MCA can be used to differentiate between specific PCR products of *CHD-Z *and *CHD-W *genes, genes vary among species and the difference in length of the PCR products of *CHD-Z *and *CHD-W *in some cases will be too close to allow for precise sexing. For example, the length of *CHD-Z *and *CHD-W *products are too close in the case of the Black-faced Spoonbill *(Platalea minor) *[14], Tawny owl [12] and kiwi ratite *(Apteryx spp) *[15]. We found similarly close length differences in at least nine species of birds with *CHD-Z *and *CHD-W *gene records in Genbank: in the *CHD-Z *and *CHD-W *genes (accession nos. = length difference) for *Accipiter gentilis *AB096144, AB096143 (5-bp), *Milvus migrans *AB096142, AB096141 (2-bp), *Circaetus gallicus *AY313610, AY313609 (9-bp), *Spizaetus nipalensis *AB096150, AB096149 (8-bp), *Gyps indicus *DQ156155, DQ156156 (6-bp), *Gyps bengalensis *DQ156153, DQ156154 (5-bp), *Accipiter nisus *AB096152, AB096151 (4-bp), *Circus spilonotus *AB096146, AB096145 (4-bp), and *Aquila chrysaetos *AB096148, AB096147 (3-bp), respectively. Therefore, a difference between the bands would be too difficult to resolve using agarose gel electrophoresis. Alternatively, polyacrylamide gels may provide sufficient resolution to discriminate between *CHD-Z *and *CHD-W *products for some species [12,15]. Yet, it is still time-consuming and it makes high-throughput screening of avian molecular sexing more difficult.

In this study, two different species of birds, *Spilornis cheela hoya *and *Pycnonotus sinensis*, were chosen as examples to evaluate the P2/P8 primer and a redesign of the sex-specific primers for *CHD-Z *and *CHD-W *genes of *S. c. hoya *using real-time PCR/MCA. Overall, the aim of this study was to develop a rapid and high-throughput SYBR green-based real-time PCR assay that would distinguish male and female birds via real-time PCR and unique melting curve profiles.

## Methods

### Sample collection and blood DNA extraction

A scientific and banding permit number 96YULI032 was issued by the Council of Agriculture of Taiwan pursuant to the Wildlife Conservation Law of Taiwan. Thirteen banded Formosan Crested Serpent Eagles *(Spilornis cheela hoya, S. c. hoya)*, numbered Bd6 and Bds12~24, were collected with official permission from the Kenting National Park (21°55' N and 120°49' E), Taiwan. Bd6 and Bd15 of *S. c. hoya *were used for the female positive control of molecular sexing based upon their behavior of playing parts in incubation, brooding and feeding. Two Chinese Bulbuls *(Pycnonotus sinensis, P. sinensis) *numbered 3424 (female) and 927 (male) were provided by the Taiwan Endemic Species Research Institute. Blood DNA was extracted immediately after sample collection using a Qiagen blood kit and stored at -20°C until needed.

### PCR and primary molecular sexing by P2/P8 primers

The universal P2/P8 primer pair [12] for avian molecular sexing was used for *S. c. hoya *and *P. sinensis *species. The primer sequence information is: forward primer P2 (5'-TCTGCATCGCTAAATCCTTT-3') and the reverse primer P8 (5'-CTCCCAAGGATGAGRAAYTG-3'). DNA samples were added to a modified PCR reaction mixture (10 μl) containing 1× PCR buffer, 1.5 mM MgCl_2_, 0.2 mM dNTPs, 0.7U Taq enzyme (Invitrogen corp.), 0.16 μM primers mix (1:1), and 10–20 ng DNA. The PCR program was followed: 94°C (4 min); 5 cycles of 94°C (30 s), 47°C (30 s), 72°C (30 s); 49 cycles of 94°C for (30 s), 46°C (20 s), 72°C (20 s); and 72°C (5 min).

### TA cloning and nucleotide sequence analysis for CHD-Z and CHD-W genes

**Figure 1:**
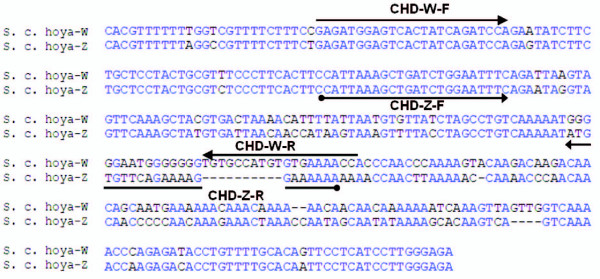
Alignment of *CHD-W *and *CHD-Z *sequences from *S. c. hoya*. Sequences are derived from the complementary version of their Genbank accession numbers DQ885237 and DQ885238, respectively.

For *S. c. hoya*, P2/P8 PCR products were gel isolated (MiniElute gel extraction kit, QIAGEN), cloned (pGEM -T Easy TA Cloning, Promega) and sequenced. The female and male birds were selected for *CHD-W *and *CHD-Z *abundant sources because the *CHD-Z *and *CHD-W *PCR products were hard to resolve in regular agarose gel. The sequencing results for the P2/P8 PCR products of *S. c. hoya *are shown in Fig. 1. For *P. sinensis*, the *CHD-Z *and *CHD-W *PCR products were easy to resolve in regular agarose gel without TA cloning technique. The length difference between *CHD-Z *and *CHD-W *PCR products using P2/P8 primers is 13- and 52-bp for *S. c. hoya *and *P. sinensis*, respectively.

### Secondary molecular sexing

An alignment performed with Biology Workbench 3.2 at the San Diego Supercomputer Center (SDSC) [16] is shown in Fig. 1. The primers for *CHD-Z- *and *CHD-W-*specific PCR of *S. c. hoya *were redesigned as follows: *CHD-W*-F: 5-GAGATGGAGTCACTATCAGATCC-3, *CHD-W*-R: 5-GGTTTTCACACATGGCACA-3; *CHD-Z*-F: 5-CATTAAAGCTGATCTGGAATTTC, *CHD-Z*-R: 5-TTTTTTCCTTTTCTGAACACATATTT-3. The lengths for PCR products amplified by *CHD-W*-F/*CHD-W*-R and *CHD-W*-F/*CHD-W*-R are 185- and 112-bp, respectively. The same PCR conditions as described above were used and the PCR program was modified: denaturation for 3 min at 95°C followed by 45 cycles of denaturation at 95°C for 30 s; annealing at 58°C for 30 s; and extension at 72°C for 20 s.

### Real-time PCR and melting curve analysis (MCA)

Real-time PCR was performed using MyiQ (Bio-Rad Laboratories, Hercules, CA). The programs for each PCR reaction, including P2/P8 primers for *S. c. hoya *and *P. sinensis*, and *CHD-Z- *and *CHD-W-*specific primers for *S. c. hoya*, are described above. After completion of the PCR reaction, a melting curve was recorded by holding at 95°C for 1 min, cooling to 55°C 1 min, and then heating slowly at 0.5°C/s until 95°C under maximal ramp rate by default in MyiQ instrument (80 repeats of counts). The melting peaks were plotted by the -dF/dT versus T formula (F is fluorescence; T is temperature).

### Control analysis of amplification product

To confirm the presence and purity of amplicons, PCR products were resolved on a 4% agarose gel, stained with ethidium bromide, and photographed.

## Results

### Molecular sexing of S. c. hoya and P. sinensis using P2/P8 primers and its gel electrophoresis

**Figure 2:**
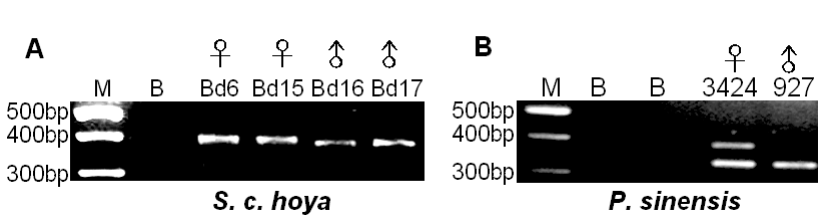
Gel view for PCR product amplified by primer sets P2/P8 in *S. c. hoya *and *P. sinensis*. Bds 6, 15, and 3424 are the female positive control. M, 100-bp marker; B-blank.

To test the feasibility of the P2/P8 primers in sexing of *S. c. hoya *and *P. sinensis*, we first performed a regular PCR reaction using this primer pair followed by gel electrophoresis, as shown in Fig. 2. For *S. c. hoya*, the *CHD-W *and *CHD-Z *gene products were unsolved in 3% agarose gel (Fig. 2A). After cloning as described in Materials and Methods, the *CHD-W *and *CHD-Z *genes of *S. c. hoya *were sequenced and submitted to Genbank with accession nos. DQ885238 and DQ885237, respectively. The difference in length (13-bp) between these *CHD-W *and *CHD-Z *genes of *S. c. hoya *was too short to resolve in 4% agarose gel. In contrast, in the case of *P. sinensis*, two distinct PCR products corresponding to *CHD-Z *and *CHD-W *genes were amplified and could be resolved in regular agarose gels (Fig. 2B). These two bands—i.e. *CHD-W *and *CHD-Z *gene products—were purified, sequenced and submitted to Genbank with accession nos. EF582413 and EF582412, respectively. There is a 52-bp length difference between the *CHD-W *and *CHD-Z *genes of *P. sinensis*. Accordingly, the P2/P8 primer set is informative for molecular sexing of *P. sinensis *but it is not suitable for the sex-specific primers for *S. c. hoya*. In short, the gender identification of *S. c. hoya *needs to be improved.

### Real-time PCR/MCA of S. c. hoya and P. sinensis using P2/P8 primers

**Figure 3:**
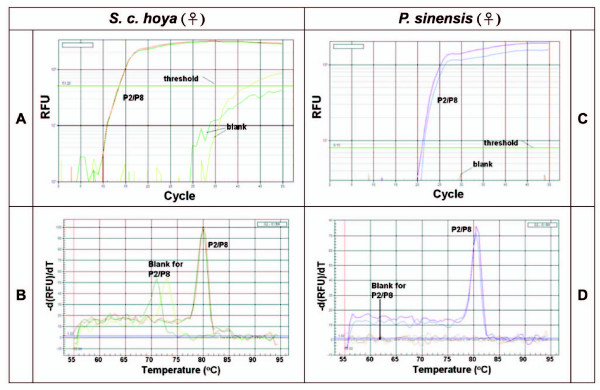
Real-time PCR/MCA using primer sets P2/P8. DNAs were chosen from *S. c. hoya *sample Bd6 (A, B) and *P. sinensis *sample 3424 (C, D), which are the ecology female control. (A, C) Real-time PCR data. (B, D) Data of MCA. The Tm of P2/P8 PCR products of *S. c. hoya *and *P. sinensis *is about 80.0 and 80.5°C, respectively. These data are collected from duplicated experiments. RFU, Relative fluorescence unit.

Real-time PCR analyses for *S. c. hoya *(Fig. 3A) and *P. sinensis *(Fig. 3C) demonstrated that the PCR reactions were successful. Reaction products were also confirmed by gel electrophoresis (data not shown). However, SYBR green-fluorescence of PCR products for *CHD-Z *and *CHD-W *genes were counted totally rather than individually. Consequently, the real-time PCR results cannot clearly provide evidence that *CHD-Z *or/and *CHD-W *genes are present individually or are present in both of them.

After performing MCA on the PCR products of *CHD-Z *and *CHD-W *genes of *S. c. hoya *amplified by P2/P8 primers in the same well, we found that there was a single peak (Tm) at 80°C for the P2/P8 PCR products in *S. c. hoya*, which could be distinguished from a nonspecific amplification with Tm at 72-74°C (Fig. 3B). The nonspecific PCR products were the primer-dimers confirmed by gel electrophoresis (data not shown). To our surprise, only a single peak at 80.5°C was found in *P. sinensis *for P2/P8 amplification (Fig. 3D) although the length difference of *CHD-Z *and *CHD-W *was 52-bp and two distinct bands were visualized (as shown in Fig. 2B). Therefore, it is unsuitable to perform SYBR green-based real-time PCR with MCA by P2/P8 primers in the example of *S. c. hoya *and *P. sinensis*.

### Redesign of sex-specific primers in S. c. hoya

In order to improve molecular sexing using real-time PCR and melting curve assay, we redesigned the sex-specific primers. In the example of *S. c. hoya*, we aligned *CHD-Z *and *CHD-W *gene sequences from Genbank accession nos. DQ885238 and DQ885237 and designed sex-specific primers for both of them (Fig. 1). The PCR products of *CHD-Z *and *CHD-W *genes are 185-bp and 112-bp, respectively. This elongated length difference between each of them was designed to make it more suitable for melting curve assay. Since the primer sets for *CHD-Z *and *CHD-W *genes overlapped, it was not suitable to perform PCR reactions in the same PCR-well (data not shown).

### Validation of sex-specific primers in S. c. hoya

**Figure 4:**
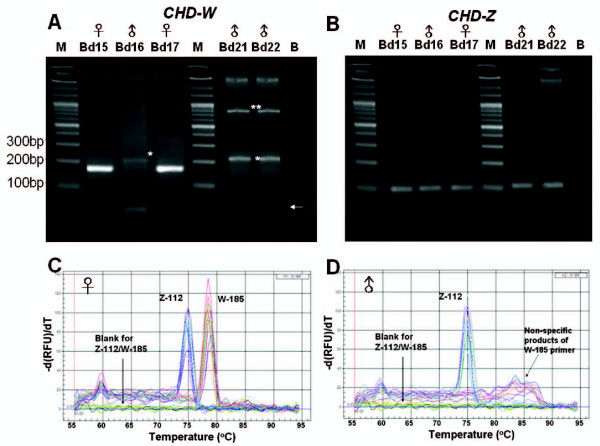
Representative gel view and MCA using sex-specific primers of *S. c. hoya*. PCR products with 185- and 112-bp amplified by (A) *CHD-W*-F/R and (B) *CHD-Z*-F/R primers in different PCR-wells were run in 4% agarose gel, respectively. The arrow of Fig. 4A indicates the remaining primers or primer-dimers of PCR. Non-specific products of W-185 indicated with star symbols were only occurred in male samples. (C, D) demonstration for high-throughput molecular sexing of multiple *S. c. hoya *samples using MCA. All data are performed in duplicate. (C) 7 females (Bds 12, 14, 15, 17, 19, 20, and 23) and (D) 6 males (Bds 13, 16, 18, 21, 22, and 24) were included. Z-112 and W-185 represent the primer mixtures for *CHD-Z*-F/*CHD-Z*-R (112-bp) and *CHD-W*-F/*CHD-W*-R (185-bp). The Tm value for Z-112 and W-185 is 75.0°C and 79°C, respectively. The Tm of non-specific products of W-185 is greatly larger than 79°C.

To examine the effect of sex-specific primers on the molecular sexing by our protocol, we performed PCR reactions using *CHD-Z *and *CHD-W *primers in different PCR-wells. With our protocol, the female bird (ZW genotype) is positive for both PCR reactions using *CHD-Z*-F/R and *CHD-W*-F/R primers. However, the male bird (ZZ genotype) is positive only for PCR reactions using *CHD-Z*-F/R primers and not for PCR reactions using *CHD-W*-F/R primers. For instance, the predicted female birds named Bd15 and Bd17 showed a single band for both *CHD-W *(Fig. 4A) and *CHD-Z *(Fig. 4B) PCR products with correct size 185-bp and 112-bp, respectively. For predicted male birds, the Bd16, Bd21 and Bd22 showed a single band of *CHD-Z *PCR products with the correct size of 112-bp (Fig. 4B). In this case, the male birds showed some weak bands for non-specific PCR products and primer-dimers using *CHD-W*-F/R primers (Fig. 4A). The size of these non-specific PCR products was not unlike that of *CHD-W*-F/R primers. Some of these bands (marked with a star symbol) were sequenced and found to be unrelated to *CHD *genes by BLAST analysis (data not shown).

### High-throughput molecular sexing of S. c. hoya- Real-time PCR/MCA

As real-time PCR is inherent with the high-throughput feature, we further evaluated the potential of our protocol for high-throughput avian molecular sexing by performing real-time PCR reactions on 13 different samples simultaneously. As shown in Fig. 4C, seven different individuals of *S. c. hoya *were identified to be females, as they exhibited both *CHD-Z- *and *CHD-W*-specific peaks at 75°C (Z-112) and 79°C (W-185), respectively. On the other hand, six different individuals of *S. c. hoya *were identified as males, as only the *CHD-Z-*specific peak at 75°C was shown in Fig. 4D. Furthermore, it was noted that the non-specific products (W-185) amplified by *CHD-W*-specific primers were discriminate to the *CHD-Z*- and *CHD-W*-specific products because of the wrong Tm value (Fig. 4D). Taken together, although only thirteen samples of *S. c. hoya *were tested in this study, our results clearly demonstrate the feasibility of our protocol for high-throughput molecular sexing of birds. 4). Further details on all the tested samples shown in Fig. 4 are shown individually in the supplementary information (see additional file 1).

## Discussion

### Application of real-time PCR/MCA for traditional PCR-electrophoresis methods using CHD gene-related primers

P2/P8 [12], 1237L/1272H [17] and 2550F/2718R [18] primer sets have been reported in the molecular sexing of birds. However, they no study has used melting curve asssay for high-throughput molecular sexing. Theoretically, MCA is able to provide the Tm value for each PCR product even if different PCR products are mixed in the same well. The use of MCA eliminates the necessity for agarose gel electrophoresis because the melting temperature (Tm) of the specific amplicon is analogous to the detection of an electrophoretic band. In this study, we focused on the issue in the case of P2/P8 primers. The fact that MCA cannot detect the individual PCR products of *CHD-Z *and *CHD-W *genes by P2/P8 primers could be due to the short length difference (13-bp for *S. c. hoya*) between these two DNA fragments (Fig. 3B).

Recently, the lengths of *CHD-W *and *CHD-Z *genes of forty-four avian species have been reported [19]. After calculation, we found that the average length difference of *CHD-W *and *CHD-Z *genes using P2/P8 primers among these species is about 40-bp (n = 44, ranging from 10- to 80-bp). Here, we chose *P. sinensis *as an example to mimic the most common PCR length difference of *CHD-Z *and *CHD-W *genes using P2/P8 primers. Its PCR length difference is 52-bp but two peaks with different Tm values were still not shown by MCA (Fig. 3D). Perhaps 52-bp is still too short for resolution in melting curve assays for *CHD-W *and *CHD-Z *genes of *P. sinensis*. In addition, the base composition and the concentration of SYBR green may play a role in Tm value and the resolution of MCA [8]. More importantly, MCA is also dependant on base pair positioning and juxtapositioning. It is possible that MCA using P2/P8 primers cannot be applied to the sexing of most bird species. In fact, our data indicated the limitations of P2/P8 primers, underlying the necessity of sex-specific primers for avian molecular sexing. Accordingly, we chose *S. c. hoya *as the example to evaluate the effect of sex-specific primers (Fig. 1) in avian molecular sexing by SYBR green-based real-time PCR followed by MCA. However, these melting curve assays were performed by a MyiQ real-time PCR machine (Bio-rad). We cannot exclude the possibility that some brands of real-time PCR machines may have different resolution. Also, high resolution melting (HRM) analysis [20,21] may resolve subtle differences even with short length differences.

### Improvement for real-time PCR combined with MCA

Using our redesigned sex-specific primers *CHD-Z*-F/*CHD-Z*-R and *CHD-W*-F/*CHD-W*-R of *S. c. hoya*, we calculated a 73-bp length difference between two PCR products (Fig. 1). This length difference proved to be long enough for resolution by MCA as shown in the results of Fig. 4. Furthermore, this gender identification method had validated by anatomical inspection (see additional file 2). However, it is possible that cross-interference of four primers mixed together in the same well hampered optimal PCR reaction (data not shown). Therefore, PCR reaction amplified by *CHD-Z*-F/*CHD-Z*-R and *CHD-W*-F/*CHD-W*-R was analyzed in different PCR-wells. This strategy proved to be successful (Figs. 4A and 4B) to determine the gender of *S. c. hoya *in a high-throughput manner (Figs. 4C and 4D).

### Nonspecific peaks in MCA

Nonspecific amplification is mainly caused by nonspecific primers but sometimes may also be due to a low annealing temperature and high concentrations of template DNA, Mg^2+^, polymerase, or dNTPs [22]. Fortunately, in our study the Tm shift derived from the PCR product polymerization can be distinguished from nonspecific amplification. Accordingly, the nonspecific amplifications have some distinct peaks in the melting curves and show some bands with different MWs from PCR products of *CHD-Z *and *CHD-W *genes (Figs. 4B and 4D).

### Other methods for molecular avian sexing

Increasing evidence [15,18,23-25] shows that the gender of some avian species cannot be identified by the P2/P8 PCR-based protocol [12] due to the limited difference in the length of intron for *CHD-Z *and *CHD-W*. This limitation has been overcome by some methods such as redesigned PCR primers [18] and our proposed approach, as well as PCR-RFLP [23,25], RAPD [26] and AFLP [27] fingerprintings. Some of these methods may also have the potential for high-throughput application of avian molecular sexing, but their gel electrophoresis step has to be improved. In contrast, our proposed PCR/MCA method is gel-free and only an extra SYBR green I is required compared to a regular PCR cocktail.

## Conclusion

Real-time PCR using SYBR green fluorescence followed by melting temperature determination is a simple and effective way to identify the gender of birds. The essential condition for this powerful molecular sexing method is the presence of *CHD-Z *and *CHD-W *sequences allowing the design of sex-specific primers. This work serves as a novel model for the development and use of a one-step, SYBR green-based real-time PCR and melting curve procedure for the rapid and specific detection and verification of the gender of birds using sex-specific or P2/P8 primer sets. Due to its real-time nature, 96- or 384-well plates are available for the real-time PCR machine to perform molecular sexing of birds in a high-throughput manner.

## Abbreviations

NCBI – National Center for Biotechnology Information; BLAST – Basic Local Alignment Search Tool; MCA – melting curve assay; CHD – chromo-helicase-DNA binding protein

## Authors' contributions

H-WC and C-C Cheng designed the experiment and wrote the manuscript. C-AC and D-LG worked on melting curve assays. C-C Chang improved the real-time PCR performance. S-HS, C-HW, Y-CC worked on DNA extraction and bioinformatics. T-CC, C-TY and, C-LT provided the samples and ecological information. All authors had read and approved the final manuscript.

## Supplementary Material

Additional file 1 Individual profile of melting curve analyses in Fig. 4. All the tested samples shown in Fig. 4 are shown individually in two grouped graphs. Click here for file

Additional file 2 Validation the results of our proposed PCR/MCA method by anatomical inspection. The anatomical structure and molecular gender identification of female samples of *S. c. hoya *were presented. Click here for file
