# Supplementary material for: High-throughput avian molecular sexing by SYBR green-based real-time PCR combined with melting curve analysis
Source: BMC Biotechnol. 2008 Feb 12;8:12. doi: 10.1186/1472-6750-8-12 (PMC2259332; doi:10.1186/1472-6750-8-12)
Supplement: Additional file 1 — Individual profile of melting curve analyses in Fig. 4. All the tested samples shown in Fig. 4 are shown individually in two grouped graphs. [file 1472-6750-8-12-S1.pdf]

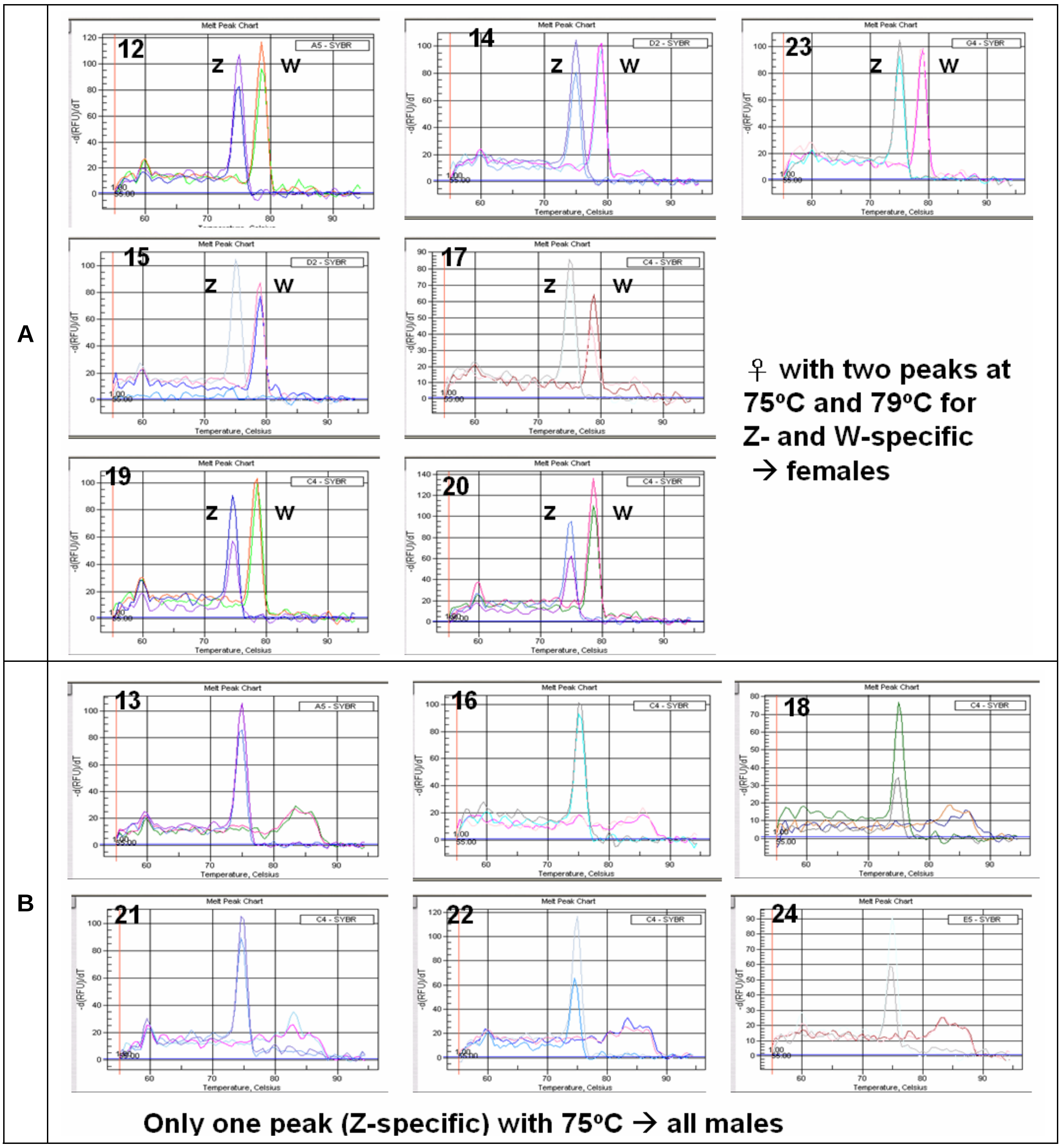


**Supplementary Fig. 1. Individual profile of melting curve analyses in Fig. 4.** All the tested samples shown in Fig. 4 are shown individually in two grouped graphs: (A) Females and (B) males. They were all performed in duplicate. The numbers in the left corner is the sample names as described as Bd no. in the Materials and methods. The figure 4 is the combined plot for these individual plots. It is depended on the selected wells in the real-time PCR analysis software. More selected wells will show more samples in the same plot. In contrast, single selected well will show only one sample.
