# Supplementary material for: High-throughput avian molecular sexing by SYBR green-based real-time PCR combined with melting curve analysis
Source: BMC Biotechnol. 2008 Feb 12;8:12. doi: 10.1186/1472-6750-8-12 (PMC2259332; doi:10.1186/1472-6750-8-12)
Supplement: Additional file 2 — Validation the results of our proposed PCR/MCA method by anatomical inspection. The anatomical structure and molecular gender identification of female samples of S. c. hoya were presented. [file 1472-6750-8-12-S2.pdf]

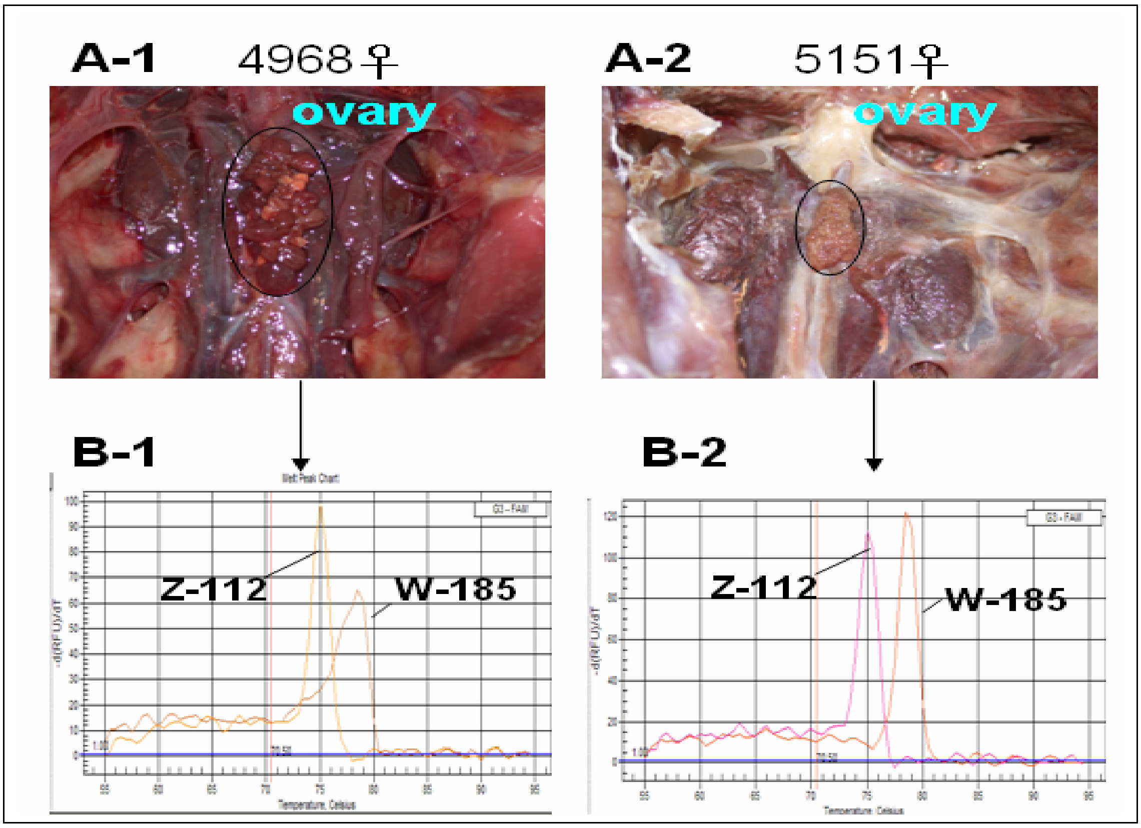

Supplementary Fig. 2. Validation the results of our proposed PCR/MCA method (A) by anatomical inspection (B). The anatomical structure and molecular gender identification of female samples (4968 and 5151) of *S. c. hoya* were presented. The sex organ ovary shown in photo was indicated by circle. In consistent with the Fig. 4, all the tested female samples are positive for both Z- and W-specific PCR.
